# The value of biomarkers in colorectal cancer: Protocol for an overview and a secondary analysis of systematic reviews of diagnostic test accuracy

**DOI:** 10.1097/MD.0000000000016034

**Published:** 2019-06-14

**Authors:** Jun Wang, Wenjia Liang, Xiangwen Wang, Guangtao Min, Wei Chen, Hongpeng Wang, Nan Yao, Jiancheng Wang

**Affiliations:** a Fourth Department of General Surgery, First Hospital of Lanzhou University; b Department of Ultrasound, Gansu Provincial Hospital; c Gansu Provincial Hospital; d Hospital Management Research Center, Lanzhou University, Lanzhou, China.

**Keywords:** biomarker, colorectal cancer, diagnostic test accuracy, overview, systematic reviews

## Abstract

**Background:**

Colorectal cancer (CRC) is the third most common cancer in the world, with 694,000 deaths each year. Despite improvements in treatment strategies in recent years, the overall survival rate of CRC is still very low and the survival rate is highly dependent on the stage at the time of diagnosis. Some biomarkers have shown great potential for early screening of CRC and some have been tested in systematic reviews (SRs). However, the quality of these SRs remains unclear and these SRs did not clarify which biomarker is the optimal diagnostic test. This overview will evaluate the methodological quality of available SRs and compare the diagnostic value of different biomarkers in order to find the best biomarker for diagnosing CRC.

**Methods:**

A comprehensive literature search for SRs published before February 2019 was conducted in the PubMed, Embase.com, Cochrane Library, and Web of Science without any language restrictions. We will use the assessment of multiple systematic reviews-2 instrument to assess the methodological quality of each SR. Bubble plots will be used to summarize the main characteristics and quality of SRs. Standard pairwise meta-analysis and adjusted indirect comparison will be conducted to compare the diagnostic value of different biomarkers.

**Results:**

The results of this overview will be submitted to a peer-reviewed journal for publication.

**Conclusion:**

The findings of this project will provide a general overview and evidence of the diagnostic value of biomarkers in detecting CRC.

**PROSPERO registration number:**

CRD42019125880.

## Abstract 2

Supplemental Digital Content is available in the text

## 1 Introduction

Colorectal cancer (CRC) is the third most common cancer in the world, with nearly 1.4 million new cases and 694,000 deaths each year.^[1,2]^ It is the fourth most common cause of cancer death in the world and the second most common cause of cancer death in the Western world.^[3–5]^ In many Asian countries, the incidence of CRC has increased 2 to 4 times over the past few decades.^[6–8]^ Despite improvements in treatment strategies in recent years, the overall survival rate of CRC is still very low.^[8]^ The survival rate of CRC depends to a large extent on the stage of the disease at the time of diagnosis.^[9]^ The 5-year survival rate for CRC patients detected at the local stage is about 90% after curative surgery, 70% for regional patients, and approximately 10% for patients with disseminated disease.^[9–11]^ Therefore, screening for rectal cancer at an early stage is essential. During the past several years, many different screening techniques have been used. However, these techniques are not ideal due to low specificity, low sensitivity, high invasiveness, or high cost.^[12,13]^ Thus, new technologies are in urgent need for CRC early detection.

In recent years, an increasing number of studies has shown that biomarkers may have great potential for early screening of CRC and some biomarkers have been tested in systematic reviews (SRs).^[14–16]^ As we know, well-conducted SRs and meta-analyses with high quality can provide the best evidence for clinical practice and healthcare decisions.^[17–19]^ If these SRs did not carry out well, it will not only waste resources but also mislead clinical practice and even cause huge losses to human health and social property. However, no studies have been conducted to evaluate the quality of these SRs. Furthermore, these SRs did not clarify which biomarker is the optimal diagnostic test for early and accurate detection of CRC. This overview will evaluate the methodological quality of available SRs and compare the diagnostic value of different biomarkers in order to find the best biomarker for diagnosing CRC.

## 2 Methods

This protocol will be reported according to the preferred reporting items for systematic reviews and meta-analysis protocols (PRISMA-P) checklist.^[20]^ As a part of our project, this protocol has been registered on the international prospective register of systematic review (PROSPERO) (CRD42019125880).

### 2.1 Selection criteria

#### 2.1.1 Type of studies

We will include SRs that reported the diagnostic value of any biomarker in diagnosing CRC. Furthermore, the SRs must meet the participants, index tests, and outcomes clarified below.

#### 2.1.2 Participants

CRC patients and the diagnosis of CRC was proven by histopathological analysis. There are no restrictions on age, race, sex, and nationality of participates, as well as treatment plan, stage and types of cancer.

#### 2.1.3 Index tests

The index tests can be a single biomarker or combined biomarkers as long as the index tests are used for detecting CRC. But combined index tests incorporate 1 biomarker and 1 imaging modality will be excluded.

#### 2.1.4 Outcome measures

SRs must explicitly state the sensitivity, specificity, diagnostic odds ratio (DOR) and their 95% confidence interval (95%CI) of biomarkers for each included original study. Or report the true positive, false positive, true negative, and false negative values for each original study to allow us to calculate the sensitivity and specificity.

#### 2.1.5 Exclusion criteria

(1) SRs did not perform the meta-analysis.
(2) SRs that did not report the diagnostic value of biomarkers which allow us to conduct quantitative analysis.
(3) SRs did not include inclusion/exclusion criteria and adequate search strategy.
(4) Conference abstracts, review articles, guidelines, consensus, documents or expert position papers, comments, letters, brief reports, proceedings, or protocol studies.

### 2.2 Literature search

The search strategies for relevant SRs were discussed by the review team and were established in co-operation with an experienced medical information specialist.^[21]^ A comprehensive literature search for SRs published before February 2019 was conducted in the PubMed, Embase.com, Cochrane Library, and Web of Science without any language restrictions. The references of included SRs were also manually retrieved for additional relevant studies. The search strategy of the PubMed is presented in Supplementary 1.

### 2.3 Selection of studies

The identified records were imported into EndNote X8 (Thomson Reuters (Scientific) LLC Philadelphia, PA) for management. After removing duplicated records, each study was screened by 2 reviewers independently based on titles and the abstracts. Any potentially eligible study was retrieved to obtain the full text for further evaluation. Then, all potential studies were evaluated independently by the same 2 authors according to the inclusion/exclusion criteria. Any disagreement was resolved by full discussion until a consensus was reached.

### 2.4 Data extraction and management

A predefined extraction form with detailed written instructions will be created using Microsoft Excel 2013 (Microsoft Corp, Redmond, WA, www.microsoft.com) to collect relevant information. Data extraction will be piloted on 10 studies to assess the reliability of data extraction across different reviewers. Then, 2 independent investigators will extract data from included SRs including general information, sample size, baseline characteristics, index tests, reference standard, and outcomes of the original study. The detailed information is presented in Table 1. If we identify multiple reviews addressing the same research question but share the same primary study, the data of the overlapping original studies will be included only once. If the same author reported their results acquired from the overlapping people or multiple published data in different original articles, only the most complete paper containing the most information is included. We will contact study authors for missing or unclear data. Disagreements on extractions will be resolved by discussion.

**Table 1:**
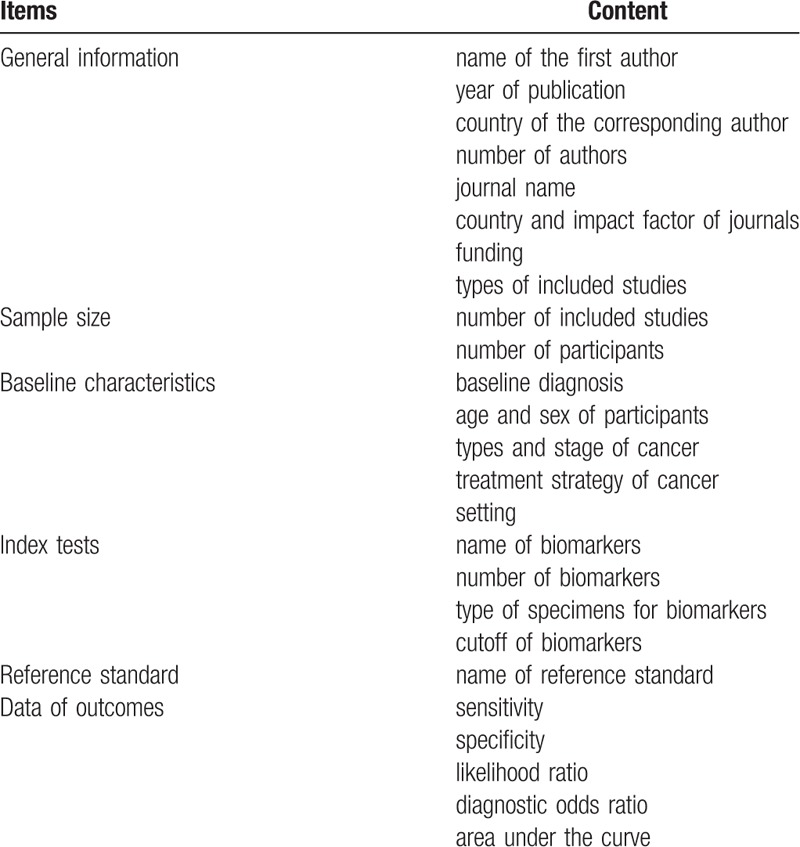
Items of data extraction.

### 2.5 Assessment of methodological quality

To assess the methodological quality of each SR, 2 independent investigators will use the assessment of multiple systematic reviews-2 (AMSTAR-2) instrument for assessment. AMSTAR-2, the updated version of original AMSTAR, can be used to appraise SRs of randomized and nonrandomized studies of health care interventions.^[22–25]^ It contains 16 items, among which 7 are critical domains, and each item will be judged as “Yes,” “No,” or “Partial Yes.” The overall quality of each SR will be explicitly classified as high, moderate, low, and critically low according to the critical domains. Discrepancies between reviewers will be resolved by discussion or by a third reviewer if consensus cannot be reached.

### 2.6 Data analysis

#### 2.6.1 Evidence map

We will create a bubble plot to summarize the main characteristics and quality of each SR. The size of the bubble will represent the number of participants, the color of the bubble will represent the methodological quality of SRs, the *x*-axis will denote the name of biomarkers, and the *y*-axis will reveal the number of SRs.

#### 2.6.2 Pairwise meta-analysis

The pairwise meta-analysis will be conducted with STATA (13.0; Stata Corporation, College Station, TX) using the data of sensitivity, specificity, DOR, positive likelihood ratio, negative likelihood ratio, and their 95%CI lower limit, 95%CI upper limit extracted from each original study of the SRs. The analysis will be performed using the Mantel–Haenszel statistical method with a random-effects model. To assess the heterogeneity among studies, we will use the chi-squared test and the *I*^2^ statistics. The *I*^2^ statistics of 25%, 50%, and 75% will be considered as cut-off points for low, moderate and high degrees of heterogeneity, respectively. If we find considerable heterogeneity among the studies, we will conduct subgroup analyses to explore the sources of heterogeneity.

#### 2.6.3 Adjusted indirect comparisons

The indirect comparisons will be conducted with the data of relative sensitivity, relative specificity, and relative DOR between different biomarkers calculated by STATA (13.0; Stata Corporation).

#### 2.6.4 Subgroup analysis

We will identify all the primary studies that reported results of subgroup analysis and extract data from these studies. If there is enough data available from the primary research. We will perform subgroup analyses according to the sex, age, and weight of patients; the country of the study; and the cutoff of biomarkers.

#### 2.6.5 Assessment of publication bias

Potential publication bias will be assessed using the Egger test and funnel plot for results with greater than or equal to 10 studies.

## 3 Results of study selection

**Figure 1:**
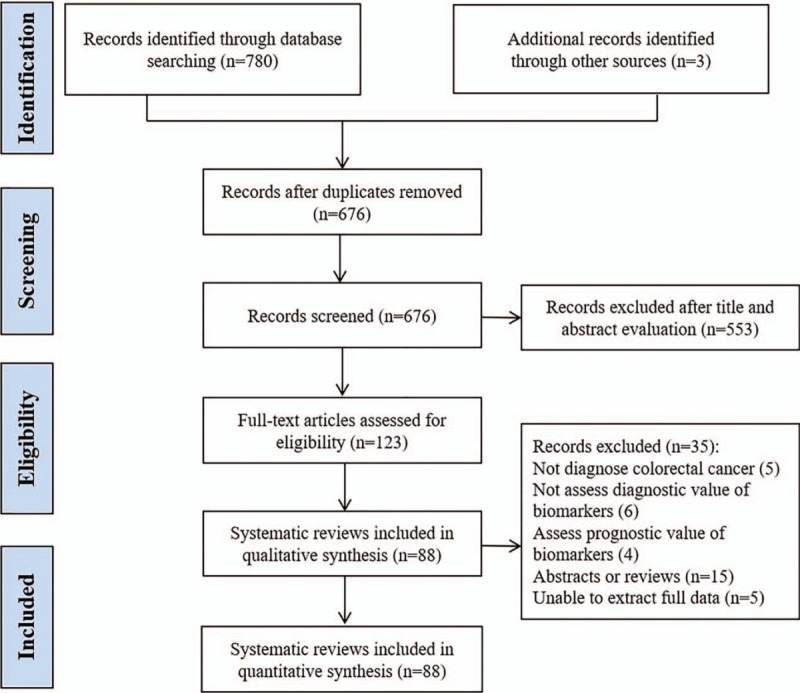
PRISMA flow chart of literature section. PRISMA = preferred reporting items for systematic review and meta-analysis.

The electronic searches identified 783 potentially relevant records, among which 780 identified through database searching and 3 identified through the manual screening. After reviewing the titles and abstracts, 553 records were excluded and 123 SRs were selected for retrieve full-texts. After full-text evaluation, 88 SRs were entered in the quantitative synthesis. Among the remaining 35 excluded SRs, 5 were not on CRC, 6 were not assessed the diagnostic value of biomarkers, 4 were assessed the prognostic value of biomarkers, 15 were abstracts or reviews, and 5 SRs failed to extract detailed data. The PRISMA flow chart of literature section is presented in Figure 1.

## 4 Ethics and dissemination

Ethical approvals and patient consent are not necessary because this is an overview based on published SRs. The findings of this project will provide a general overview and evidence of the diagnostic value of biomarkers in detecting CRC. The results will be submitted to a peer-reviewed journal for publication. We hope that these findings will help clinicians and patients choose a more appropriate method for rectal cancer diagnosis.

## Author contributions

JW, WJL, and NY planned and designed the research. XWW, GTM, WC, HPW, and JCW tested the feasibility of the study. JW, WJL, and NY and provided methodological advice, polished and revised the manuscript. JW and NY and wrote the manuscript. All authors approved the final version of the manuscript.

**Conceptualization:** Jun Wang, Wenjia Liang, Nan Yao.

**Data curation:** Jun Wang, Wenjia Liang, Xiangwen Wang, Guangtao Min.

**Formal analysis:** Jun Wang, Nan Yao.

**Funding acquisition:** Nan Yao, Jiancheng Wang.

**Investigation:** Wenjia Liang, Xiangwen Wang, Guangtao Min, Wei Chen, Hongpeng Wang.

**Methodology:** Jun Wang, Nan Yao.

**Project administration:** Nan Yao.

**Resources:** Xiangwen Wang, Guangtao Min, Wei Chen.

**Software:** Jun Wang, Wenjia Liang, Hongpeng Wang.

**Supervision:** Nan Yao.

**Validation:** Nan Yao, Jiancheng Wang.

**Visualization:** Xiangwen Wang, Guangtao Min, Wei Chen.

**Writing – original draft:** Jun Wang, Wenjia Liang, Nan Yao.

**Writing – review and editing:** Jun Wang, Nan Yao.

## Supplementary Material

Supplemental Digital Content
