# Supplementary material for: The value of biomarkers in colorectal cancer: Protocol for an overview and a secondary analysis of systematic reviews of diagnostic test accuracy
Source: Medicine (Baltimore). 2019 Jun 14;98(24):e16034. doi: 10.1097/MD.0000000000016034 (PMC6587652; doi:10.1097/MD.0000000000016034)
Supplement: Supplemental Digital Content [file medi-98-e16034-s001.docx]

**The search strategy of PubMed**

#1 Colorectal Neoplasms[Mesh] OR colorectal neoplasm*[Title/Abstract] OR colorectal tumor*[Title/Abstract] OR colorectal carcinoma*[Title/Abstract] OR colorectal cancer*[Title/Abstract]

#2 Rectal Neoplasms[Mesh] OR rectal neoplasm*[Title/Abstract] OR rectal tumor*[Title/Abstract] OR rectal tumour*[Title/Abstract] OR rectal carcinoma*[Title/Abstract] OR rectal cancer*[Title/Abstract] OR rectum neoplasm*[Title/Abstract] OR rectum tumor*[Title/Abstract] OR rectum tumour*[Title/Abstract] OR rectum carcinoma*[Title/Abstract] OR rectum cancer*[Title/Abstract]

#3 Colonic Neoplasms[Mesh] OR colon neoplasm*[Title/Abstract] OR colon tumor*[Title/Abstract] OR colon tumour*[Title/Abstract] OR colon carcinoma*[Title/Abstract] OR colon cancer*[Title/Abstract] OR colonic neoplasm*[Title/Abstract] OR colonic tumor*[Title/Abstract] OR colonic tumour*[Title/Abstract] OR colonic carcinoma*[Title/Abstract] OR colonic cancer*[Title/Abstract]

#4 Adenomatous Polyposis Coli[Mesh] OR adenomatous intestinal polyposes[Title/Abstract] OR adenomatous intestinal polyposis[Title/Abstract] OR adenomatous polyposis colus[Title/Abstract] OR adenomatous polyposis coli[Title/Abstract] OR familial adenomatous polyposes[Title/Abstract] OR familial adenomatous polyposis[Title/Abstract] OR familial intestinal polyposes[Title/Abstract] OR familial intestinal polyposis[Title/Abstract] OR familial multiple polyposes[Title/Abstract] OR familial multiple polyposis[Title/Abstract] OR familial multiple polyposus[Title/Abstract] OR familial polyposis coli[Title/Abstract] OR familial polyposis colus[Title/Abstract] OR familial polyposis syndrome[Title/Abstract] OR familial polyposis syndromes[Title/Abstract] OR hereditary polyposis coli[Title/Abstract] OR hereditary polyposis colus[Title/Abstract] OR myh associated polyposes[Title/Abstract] OR myh associated polyposis[Title/Abstract] OR myh-associated polyposes[Title/Abstract] OR myh-associated polyposis[Title/Abstract] OR polyposis coli[Title/Abstract] OR polyposis colus[Title/Abstract]

#5 #1 OR #2 OR #3 OR #4

#6 "Biomarkers, tumor"[Mesh]

#7 cancer biomarker*[Title/Abstract] OR cancer bio-marker[Title/Abstract] OR cancer marker*[Title/Abstract] OR tumor biomarker*[Title/Abstract] OR tumor bio-marker[Title/Abstract] OR tumor marker*[Title/Abstract] OR tumour biomarker*[Title/Abstract] OR tumour bio-marker[Title/Abstract] OR tumour marker*[Title/Abstract] OR carcinogen biomarker*[Title/Abstract] OR carcinogen bio-marker[Title/Abstract] OR carcinogen marker*[Title/Abstract] OR neoplasm biomarker*[Title/Abstract] OR neoplasm bio-marker[Title/Abstract] OR neoplasm marker*[Title/Abstract] OR neoplasm metabolite marker*[Title/Abstract] OR tumor metabolite marker*[Title/Abstract] OR cancer metabolite marker*[Title/Abstract] OR tumour metabolite marker*[Title/Abstract] OR carcinogen metabolite marker*[Title/Abstract]

#8 #6 OR #7

#9 "Sensitivity AND Specificity"[Mesh] OR "False Positive Reactions"[Mesh] OR "False Negative Reactions"[Mesh] OR "ROC Curve"[Mesh] OR "Predictive Value of Tests"[Mesh]

#10 sensitivity[Title/Abstract] OR specificity[Title/Abstract] OR "receiver operating characteristic"[Title/Abstract] OR "receiver operator characteristic"[Title/Abstract] OR "predictive value*"[Title/Abstract] OR roc[Title/Abstract] OR "pre-test odds"[Title/Abstract] OR "pretest odds"[Title/Abstract] OR "pre-test probability*"[Title/Abstract] OR "pretest probability*"[Title/Abstract] OR "post-test odds"[Title/Abstract] OR "posttest odds"[Title/Abstract] OR "post-test probabilit*"[Title/Abstract] OR "posttest probabilit*"[Title/Abstract] OR "likelihood ratio*"[Title/Abstract] OR "positive predictive value*"[Title/Abstract] OR "negative predictive value*"[Title/Abstract] OR "false negative*"[Title/Abstract] OR "false positive*"[Title/Abstract] OR "true negative*"[Title/Abstract] OR "true positive*"[Title/Abstract]

#11 #9 OR #10

#11 "Meta-Analysis as Topic"[Mesh] OR "Meta-Analysis"[Publication Type]

#12 meta analysis[Title/Abstract] OR meta analyses[Title/Abstract] OR meta-analysis[Title/Abstract] OR meta-analyses[Title/Abstract] OR metaanalysis[Title/Abstract] OR metanalysis[Title/Abstract] OR met-analysis[Title/Abstract] OR metaanalyses[Title/Abstract] OR metanalyses[Title/Abstract] OR met-analyses[Title/Abstract] OR data pooling[Title/Abstract] OR data poolings[Title/Abstract] OR clinical trial overview[Title/Abstract] OR clinical trial overviews[Title/Abstract] OR systematic review[Title/Abstract] OR systematic reviews[Title/Abstract]

#13 #11 OR #12

#14 #5 AND #8 AND #11 AND #13
